# A Contemporary Review of the Genomic Associations of Coronary Artery Myocardial Bridging

**DOI:** 10.3390/genes14122175

**Published:** 2023-12-04

**Authors:** Peyton Moore, Paul Murdock, Akash Ramanathan, Mohanakrishnan Sathyamoorthy

**Affiliations:** 1 Sathyamoorthy Laboratory, Department of Medicine, Burnett School of Medicine at TCU, Fort Worth, TX 76123, USA; 2 Consultants in Cardiovascular Medicine and Science—Fort Worth, PLLC, 1121 5th Avenue, Suite 100, Fort Worth, TX 76104, USA

**Keywords:** myocardial bridging, genomics, congenital coronary vascular anomalies, single nucleotide variants

## Abstract

Background: Myocardial bridging (MB) is a congenital coronary artery anomaly that has limited molecular disease state characterization. Though a large portion of individuals may be asymptomatic, the myocardial ischemia caused by this anomaly can lead to angina, acute coronary syndrome, coronary artery disease, and sudden cardiac death in patients. Objective: This study aims to summarize and consolidate the current literature regarding the genomic associations of myocardial bridge development and, in doing so, prompt further investigation into the molecular basis of myocardial bridge development. Methods: We performed a systematic literature review of myocardial bridging using the key search terms “Myocardial Bridging” AND (“Gene” OR “Allelic Variants” OR “Genomic”) in the databases of PubMed, CINAHL, EMBASE, and Cochran. We then performed a detailed review of the resulting abstracts and a full-text screening, summarizing these findings in this report. Results: In total, we identified eight articles discussing the associated genomics behind MB development. Studies included review articles, case reports and genomic studies that led to the discussion of several genes: *DES* (E434K), *FBN1* (I1175M), and *COMMD10*; *MACROD2*, *SLMAP*, *MYH7* (A1157G), and *DPP6* (A714T); *MYH7* (A862V); *SCN2B* (E31D); and *NOTCH1* (R2313Q), and to the discussion of miRNAs (miR-29b, miR-151-3p, miR-126, miR-503-3p, and miR-645). Conclusions: Our study is the first to summarize the genes and molecular regulators related to myocardial bridges as they exist in the current literature. This work concludes that definitive evidence is lacking, warranting much broader genetic and genomic studies.

## 1. Introduction

**Figure 1:**
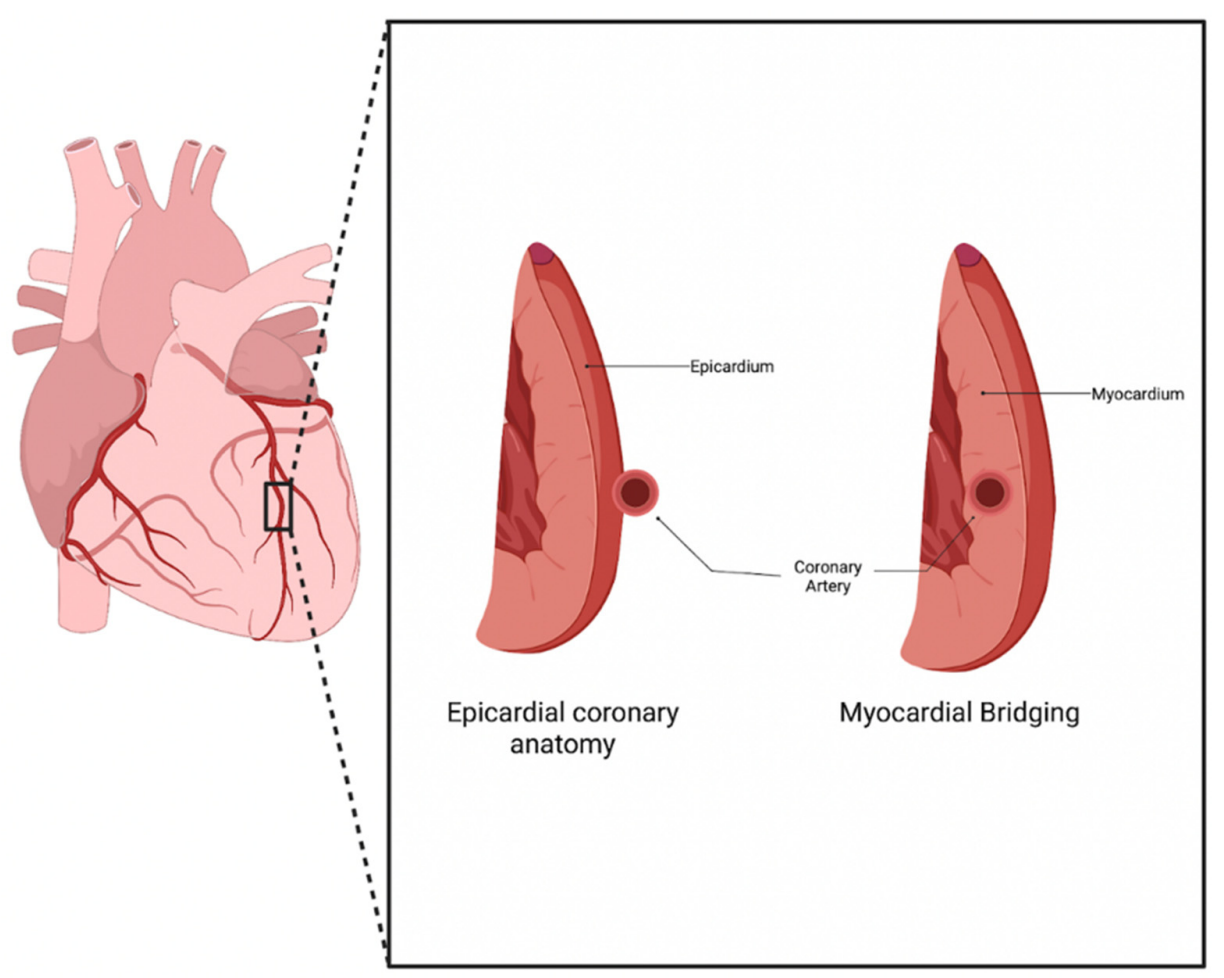
Myocardial Bridging anatomy. Normal coronary artery anatomy consists of the artery traveling along the epicardium of the heart, while a bridging artery travels within the myocardium [3].

Normal coronary artery anatomy consists of the coronary arteries arising from the aortic sinuses, which follow a converging path toward the heart’s anatomical apex. The three main coronary arteries (the right coronary artery (RCA), left anterior descending (LAD), and left circumflex (LCX)) each have common placements and travel along the epicardium of the heart in patients who underwent typical coronary development [1]. Myocardial bridging (MB) is a congenital anomaly that consists of a portion of an epicardial coronary artery diving into the myocardium layer for a section of its journey (Figure 1). The muscle layer that lies above the artery is recognized as a myocardial bridge, while the portion of the artery is labeled as a tunneled artery. This anomaly is typically seen in the LAD, but may also be present in any of the coronary arteries [2]. Our clinical experience has verified this phenotypic presentation. 

The genetic mechanisms driving the development of congenital coronary vascular anomalies (CCVAs), including those of myocardial bridging, lack sufficient research [4]. This finding is surprising as these anomalies are present and well characterized in patients throughout the world. The rates of myocardial bridging vary between the methods of evaluation, with autopsy and angiography being the most common techniques used to assess for the presence of bridging. Invasive angiography generally underreports the prevalence of myocardial bridging, as it typically detects the anomaly in 0.15–25% of patients [1]. This low prevalence is countered by autopsy reports, which illustrate the prevalence of MBs in 5–86% of patients [5]. Recent computed tomography (CT) studies have also shown bridging in up to 25% of patients, further showing the underestimation of its prevalence within angiography studies [1]. In our own CCTA cohort from 2014–2023, the prevalence is 7% (70/1000) [6]. The discrepancy in these methods demonstrates the unreliability of the current diagnostic methods and suggests a need for improved techniques.

**Figure 2:**
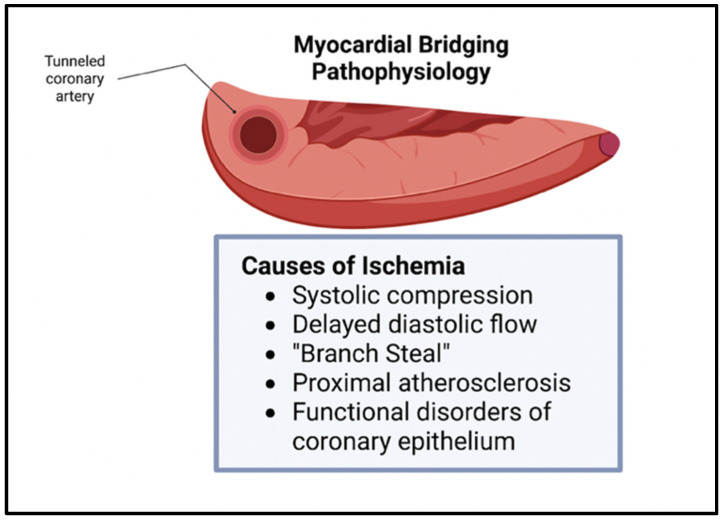
Myocardial Bridging physiology. Ischemia in myocardial bridging is secondary to systolic compression, delayed diastolic flow, branch steal, proximal atherosclerosis, and functional disorders of coronary epithelium [3].

Though bridging was previously viewed as benign, this belief has been challenged in the recent literature. The vessel compression of the intramural artery has been noted during the contraction of the myocardium, often leading to chest pain and, sometimes, to acute coronary syndrome [7]. The overarching pathophysiology behind these symptoms stems from the myocardial ischemia secondary to the tunneling and compression of the artery, with the ischemia being exacerbated by the increased sympathetic tone [2]. Since the systolic phase of the myocardial cycle is only mildly involved in the perfusion of the myocardial tissue, other mechanisms contribute to the ischemia seen in bridging. These mechanisms include delayed early diastolic artery relaxation, atherosclerotic stenosis development proximal to the tunneled artery, functional disorders of coronary circulation including impaired endothelium-dependent vasodilatation and microvascular dysfunction, and the “branch steal” phenomenon (Figure 2). The branch steal effect describes the crossing of blood through the constricted segment during the end of systole and early diastole, leading to an increase in diastolic flow velocity in the artery. The ischemia due to these various mechanisms are important causes of the anginal symptoms that bridging patients experience [8]. Other cardiovascular events linked to myocardial bridging include cardiac arrhythmias and sudden cardiac death [9].

### Objectives

Myocardial bridging is a symptomatic cardiac anomaly that is underdiagnosed in many individuals and has historically been under-researched. A few systematic reviews have been published detailing its clinical features and hypothesizing mechanisms regarding myocardial bridge development throughout the last decade, however, research into the genomic association behind bridge development remains sparse. The objective of this manuscript is to review and discuss prior discoveries related to genomics and bridge development, serving as an up-to-date reference to prompt further hypothesis-driven investigations.

## 2. Methods

### 2.1. Overview

This study used a structured and comprehensive approach that included a methodological approach for capturing articles containing research on myocardial bridging and genomic variance. Various popular scientific databases were used to gather relevant articles, and strict inclusion and exclusion guidelines were followed by each author.

### 2.2. Published Literature Search

A literature search was conducted using Pubmed, CINAHL, EMBASE, and Cochrane (Table 1). The search terms used in each of these databases are summarized in Figure 1. The terms “myocardial bridging” and “genomics” were searched in each of the listed databases. Only original, peer-reviewed, clinical research studies published in English within the last 10 years were considered. A search of the Cochrane database yielded no relevant results. Out of the articles included, several were found in more than one of the databases searched.

For inclusion in our study, the following inclusion criteria had to be met:

1. Published on or after 1 January 2013.
2. Study published in English.
3. Evidence of discussion on myocardial bridging and its association with a gene, allele variant, or genomic association.

Studies were excluded from further consideration if the exclusion criteria were met including:

1. Study published prior to 1 January 2013.
2. Study not published in English.
3. No mention of an association between myocardial bridging and genomics or genetic variance.

### 2.3. Data Extraction and Analysis

Each author conducted a search in Pubmed, CINAHL, EMBASE, and Cochrane. All authors yielded the same number of results. Out of the resultant papers, each author identified which papers to include. Each author yielded an identical list, representing congruence amongst the authors. Once the resultant papers were reviewed by each author and found to have met the inclusion criteria, a final list of resultant articles was synthesized.

From the 53 papers found across our four databases, we extracted relevant study characteristic, genomic, and biologic data from our resultant eight papers. These data included the cause of death, mutation, and subject information. These clinical and genomic variables are detailed below (Table 2).

#### Study Characteristics

**Figure 3:**
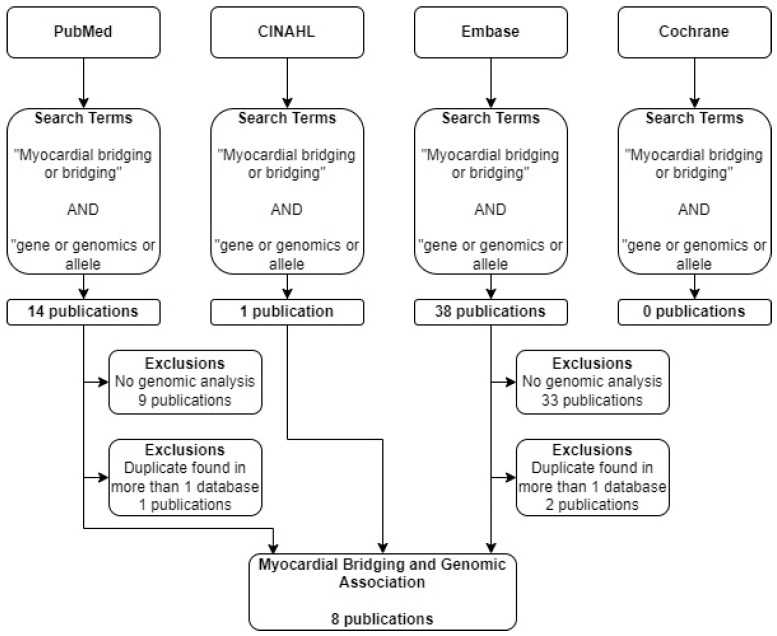
Flow diagram describing selection process of articles. Four databases were searched using identical search terms. Excluding duplicates across databases, 8 publications met selection criteria.

A total of 53 eligible articles were found using our search criteria. Out of these, eight met our inclusion criteria and involved the discussion of myocardial bridging and genomic analysis (Figure 3). Out of these, 6 were case reports (Table 2). Each of these articles and case reports were read by each author and discussed in detail.

### 2.4. Case Reports

Out of the eight resultant papers that met our inclusion criteria, six were case reports. The mean subject age was 18 and the median age 15.5. Four out of six (4/6, 66%) of the subjects were female. Two of the six (2/6, 33%) papers discussed an *MYH7* mutation. The remaining four papers reviewed other genes. Two of the six (2/6, 33%) subjects died of sudden cardiac death.

### 2.5. Research Articles

Out of the eight resultant papers that met our inclusion criteria, two were research articles. The first discussed the microRNA expression profile seen in myocardial bridge patients. The second paper discussed the cardiac manifestations of the *PRKAG2* mutation within the context of a case presentation.

## 3. Genetic Mutations Associated Myocardial Bridges: Potential Candidate Genes?

### 3.1. MYH7

ß-myosin heavy chain (*MYH7*) gene mutations have been associated with hypertrophic cardiomyopathy (HCM) and restrictive cardiomyopathy (RCM). The *MYH7* mutations found in a 9-month-old female with RCM and associated myocardial hypertrophy and a 7-year-old female who died of sudden cardiac death (SCD) were classified as variants of unknown significance (VUS). Of note, both patients were found to have myocardial bridging of the left anterior descending (LAD) artery on autopsy [10,11]. Genetic testing of one of the patients revealed an A1157G mutation in exon 13 of the *MYH7* gene. This mutation resulted in a nonsynonymous amino acid change at position 386 from tyrosine to cysteine and was classified as being likely pathogenic [10,11].

The *MYH7* gene encodes for ß-myosin heavy chain, an essential component of the cardiac sarcomere. Though the authors do not hypothesize a mechanism regarding bridge formation, there are other examples of associations between *MYH7* mutations and bridge formation including the case of a 15-year-old with exertional dyspnea and chest pain [12]. We view this as a potential candidate gene of interest for further studies, as mutations in the gene may have significant clinical manifestations outside of its already known associations with HCM and RCM.

### 3.2. DPP6

The *DPP6* gene encodes a membrane protein that is a member of the peptidase S9B family of serine proteases and is known to bind specific voltage-gated potassium channels [13]. Mutations in the *DPP6* gene have been previously associated with the development of idiopathic ventricular fibrillation (IVF), which is known to cause SCD [14]. Mutations in the *DPP6* (Ala714Thr) gene were discovered in the father and sister of a child who died from SCD and was found to have a 1.1 × 0.5 cm myocardial bridge on autopsy. Further analysis of *DPP6* and the other mutated genes in the family of the proband found that they were variants of unknown significance (VUS). Importantly, the *DPP6* mutation was not the only mutation in the child. The VUS in *MYH7, SCN2B,* and *NOTCH1* were also found. The authors hypothesize that the SCD seen in the child was likely secondary to the HCM and its subsequent role in arrhythmic death, however, they are unsure of the consequences secondary to the isolated genetic variants [11]. We suspect that the *DPP6* mutation may have contributed to arrhythmias seen in the patient, however, we have low suspicion that the mutation played a role in bridge formation.

### 3.3. SCN2B

The *SCN2B* gene encodes voltage-gated sodium channel ß2-subunits, and is involved in cell–cell adhesion and cell migration [15]. Mutations in the *SCN2B* gene have been associated with cardiac arrhythmias in humans including atrial fibrillation and Brugada syndrome [16]. Mutations in the *SCN2B* gene (Glu31Asp) were discovered in a child that died from sudden cardiac death who was found to have a myocardial bridge on autopsy [11]. No mechanism regarding the gene mutation and the development of myocardial bridging was discussed in the report. Despite this, we hypothesize that the disruption in *SCN2B*’s known role in cell migration via genetic mutation may play a role in the abnormal migration of the coronary arteries during embryogenesis, which could subsequently result in the formation of myocardial bridging. Due to its known role in this vital process, further investigation into the potential relationship between mutations in *SCN2B* and bridge development may be promising.

### 3.4. NOTCH1

*NOTCH1* encodes a cell-surface receptor that plays a role in the development of various cell and tissue types [17]. The importance of the NOTCH pathway during the development of the cardiovascular system has been extensively discussed. Notably, NOTCH has several roles during ventricular development including that of trabecular development via differentiation and the proliferation of cardiomyocytes [18]. A mutation in *NOTCH1* (Arg2313Gln) was discovered in a young female that died from SCD who was found to have a myocardial bridge on autopsy [11]. Though the authors made no direct hypothesis regarding a link between *NOTCH1* and bridge development, it is plausible that mutations in a member of the NOTCH family of genes, known players in cardiovascular development, may play a role in the development of a coronary artery anomaly such as myocardial bridging.

Studies have demonstrated that *NOTCH1* plays a pivotal role in cardiomyocyte differentiation and proliferation. The gene has been implicated in various congenital heart defects including hypoplastic left heart syndrome [19]. The core of *NOTCH1*’s role in angiogenesis is its impact on endothelial cells. The gene influences the delicate balance between tip cells and stalk cells during sprouting angiogenesis. Tip cells, located at the leading edge of growing vessels, steer vessel extension, while stalk cells support vessel elongation. *NOTCH1* activity in tip cells curtails excessive sprouting by inducing the expression of Dll4, a NOTCH ligand. This creates a feedback loop where Dll4 interacts with the neighboring endothelial cells’ NOTCH receptors, repressing their tip cell potential and promoting stalk cell characteristics, thereby maintaining vessel integrity [20]. A disruption in this balance may result in a disruption of angiogenesis.

*NOTCH1* is activated in proliferating embryonic and immature cardiomyocytes and is downregulated in the myocardium during postnatal development. NOTCH signaling in adults is activated transiently in response to myocardial injury, further suggesting that the gene is contributory to cardiac repair via angiogenesis [21,22]. Due to the many roles that the *NOTCH1* gene plays in the angiogenesis and development of the cardiovascular system, further investigation assessing its potential association in bridge development is warranted as we view *NOTCH1* to be a candidate gene of interest.

### 3.5. SLMAP

A variant of unknown significance in the *SLMAP* gene (c.599c>T) was found in a 20-year-old male that passed away from sudden cardiac death. Further autopsy revealed a 2-cm myocardial bridge that began in the left anterior descending artery. The missense mutation was found to have a very low frequency in the general population, and the mutation was found to be likely pathogenic using three in silico predictors [23]. *SLMAP* is a known cardiac membrane protein that plays a role in excitation–contraction coupling in cardiac myocytes. *SLMAP* mutations have been previously linked to cardiac conditions including the development of heart failure [24]. *SLMAP* has also been associated with the development of Brugada syndrome, a cardiac channelopathy, through the silencing of *SLMAP* by small-interfering RNA (siRNA) [25]. No relationship between *SLMAP* and angiogenesis, cell migration, or coronary artery embryogenesis were discussed, limiting our suspicion for its involvement in myocardial bridge development. Based on *SLMAP’s* role in cardiac ion channel physiology rather than processes vital to coronary artery development and maturation, we do not view this as a potential candidate gene for further studies.

### 3.6. COMMD10

Deletions in *COMMD10* were identified in a 19-year-old athlete who was experiencing syncopal episodes. The coronary CTA of the patient discovered a 20 mm bridge in the LAD. An in-depth genetic analysis was performed using array-comparative genomic hybridization (array-CGH) and whole exome sequencing. The array-CGH demonstrated a copy number variation (CNV) alteration affecting intron 5 of the *COMMD10* gene, which is responsible for modulating the activity of the cullin-RING E3 ubiquitin ligase complexes (CRL) and reducing NFK-ß activation. *COMMD10* has also been found to be expressed in the endothelial cells and smooth muscle cells of various tissues, which demonstrates a potential involvement in angiogenesis during stages of embryogenesis. The gene has also been found to be expressed in cardiac tissue, which suggests it may have a role in cardiac development. The authors of the study hypothesize that *COMMD10* plays an important role during cardiac development, which may include the development of congenital heart disorders and anomalies such as myocardial bridging [26]. Due to these findings, we agree that *COMMD10* is a candidate gene of interest and believe that further research regarding the association of the gene with myocardial bridge development is warranted.

### 3.7. MACROD2

*MACROD2* is a deacetylase involved in removing ADP-ribose from mono-ADP-ribosylated proteins and is frequently involved in patients with complex syndromes [27]. A macrodeletion of *MACROD2* was discovered in an athlete suffering from syncope who was found to have a 20 mm myocardial bridge. An array-CGH demonstrated CNVs, which may be associated with the presence of congenital heart disorders (CHD). A recent genome-wide association study that included over 4000 patients affected by CHD and 8000 controls revealed a statistically significant association between MACROD2 polymorphisms and the development of the transposition of the great vessels. Further data has shown the expression of *MACROD2* is present in human embryonic cardiac cells, further strengthening the possibility of the gene’s involvement as a transcriptional regulator in cardiomyocytes [26]. Due to its presence in human embryonic cardiac cells and its potential role as a transcriptional regulator in cardiac myocytes, the authors hypothesized its contributing role in the development of CHDs. With these findings, we agree that this may be a candidate gene of interest and support further investigation into this genotype–phenotype relationship.

### 3.8. FBN1

A heterozygous mutation in the *FBN1* gene was found on whole exome sequencing in a 12-year-old girl that was experiencing recurrent syncopal episodes after exercise. The identified change was a missense mutation of c.3535A>G resulting in an amino acid change of p.I1175M in exon 29. Further bioinformatic analysis suggested that this variant may affect the structure and function of the protein product [28]. The *FBN1* gene encodes the fibrillin-1 protein, an extracellular matrix component that regulates growth factor signaling pathways. Pathologic mutations in *FBN1* are well-established causes of Marfan syndrome and other congenital heart and vascular defects [29]. There are no established associations between *FBN1* and coronary artery development, however, with the gene’s extensive role in other cardiovascular pathologies, genes in this cascade (FBN1 and FBN2) should be further characterized in myocardial bridge development as candidate genes of interest.

### 3.9. DES

A heterozygous missense variant (c.1300G>A; p.E434K) of the *DES* gene was identified using whole-exome sequencing in a Chinese family with cardiomyopathy and sudden cardiac death. A myocardial bridge was discovered in the proband; however, none was present in the other four patients. Further genetic analysis predicted the mutation to be pathogenic, which was strengthened with its absence in 200 controls. The *DES* gene encodes for desmin, an intermediate filament protein that stabilizes sarcomeres and cell contacts in the cardiac intercalated disc [30]. We have little suspicion to believe that the isolated *DES* mutation was the causative agent for bridge development. This belief is secondary to the lack of evidence of bridging found in the proband’s siblings or parents, or in other reports.

### 3.10. PRKAG2

The PRKAG2 gene is best known for its role in Wolff-Parkinson-White (WPW) and PRKAG2 syndrome. PRKAG2 syndrome is glycogen accumulation within cardiac tissue, which clinically presents as HCM. A mutation in the PRKAG2 gene was found in a 23-year-old female with a known history of WPW and HCM, who presented after a non-ST elevation myocardial infarction. The patient underwent genetic testing which revealed a heterozygous missense Arg302Gln mutation in the PRKAG2 gene. The angiography revealed severe bridging of the LAD in the patient [31]. Though this mutation may have contributed to myocardial hypertrophy secondary to the glycogen accumulation, we do not view this as a likely candidate gene for bridging development. We believe that the associated myocardial bridge in this patient is likely secondary to the underlying PRKAG2 syndrome, and not due to a primary mutation causing abnormal coronary artery development or migration.

### 3.11. Summary of Genes

**Figure 4:**
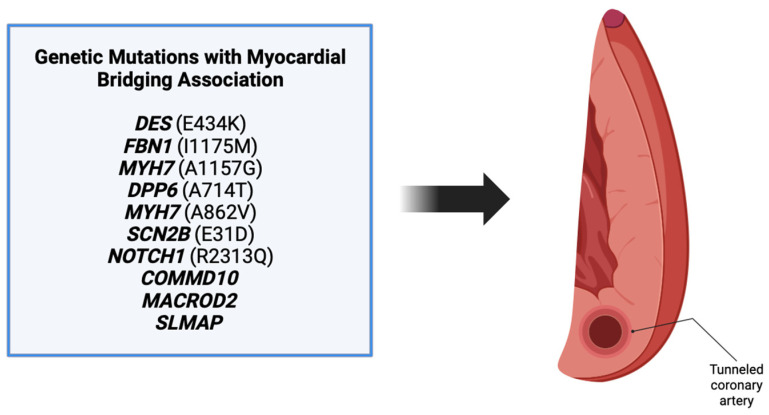
Ten genes were found associated with myocardial bridge development. These genes include *DES*, *FBN1*, *MYH7*, *DPP6*, *MYH7*, *SCN2B*, *NOTCH1*, *COMMD10*, *MACROD2*, and *SLMAP* [3].

Our review revealed a total of 10 genes in patients that were also found to have a myocardial bridging phenotype (Figure 4). Each of these genes have different roles in various processes including angiogenesis, embryogenesis, and cell differentiation, as discussed above. Patients in the cases varied in their presentation, with many being symptomatic and others having incidental findings of myocardial bridging on imaging or autopsy. Regardless of the presentation, the background of each gene was reviewed to determine its potential role in myocardial bridge development.

Of the mutations discovered in the review, we only view six to be candidate genes for further research: *MYH7*, *SCN2B*, *NOTCH1*, *COMMD10*, *MACROD2*, and *FBN1/FBN2* (Table 3). These genes have been found to be key in various processes that are vital for coronary artery development and/or migration, and it is plausible that disruptions in these genes may serve as a genesis for the development of myocardial bridging. These findings warrant further investigation into the potential mechanistic basis behind mutations in the candidate genes and the development of myocardial bridging.

## 4. Myocardial Bridging and MicroRNA Expression

MicroRNAs (miRNAs) are short, non-coding RNAs that act as post-transcriptional regulators. Prior studies have identified miRNAs as having tissue or cell-specificity, making them potential diagnostic tools. Of note, five circulating miRNAs have been identified as possible biomarkers for myocardial bridge detection. These miRNAs, miR-29b, miR-151-3p, miR-126, miR-503-3p, and miR-645, were all capable of distinguishing patients with known myocardial bridges from control patients [32]. This is an important finding that needs further exploration through a larger cohort of genomic study in CT angiographically confirmed myocardial bridge patients. If positive findings are reported, these miRNAs may serve as novel genetic biomarkers for more cost-effective myocardial bridge screening. Furthermore, use of these biomarkers may improve diagnostic accuracy in patients with myocardial bridging, as current imaging resources vary in reliability. Due to this, further investigation into the relationship between various miRNAs and myocardial bridging presence is warranted.

## 5. Conclusions

Myocardial bridging is associated with potentially life-threatening complications including acute MI, tachycardia, syncope, and sudden cardiac death. Myocardial bridges are coronary artery anomalies that are significantly underdiagnosed, and the contemporary literature focuses primarily on symptoms instead of potential genetic causes. Literature has recently emerged proposing the mechanisms behind development, however, research is very limited on identifying genetic and genomic associations. Our systematic review of the literature revealed myocardial bridge development in patients with mutations in *MYH7*, *DPP6*, *SCN2B*, *NOTCH1*, *SLMAP*, *COMMD10*, *MACROD2*, *FBN1*, *DES*, and *PRKAG2.* Of these genes listed, *MYH7*, *SCN2B*, *NOTCH1*, *COMMD10*, *MACROD2*, and *FBN1* show the most promise to be candidate genes associated with myocardial bridge development, justifying the further investigation of the mechanisms behind bridge development. Furthermore, recent literature has reported the association of myocardial bridging with specific miRNAs (miR-29b, miR-151-3p, miR-126, miR-503-3p, and miR-645), a tool that may be used for myocardial bridge detection. Additional larger studies into the presence of miRNAs and subsequent myocardial bridge detection are warranted, as these biomarkers may prove to be cost-effective methods for detecting myocardial bridging in both asymptomatic and symptomatic patients. As a definitive molecular basis for myocardial bridge development is lacking, our group has initiated the genomic investigation of a large cohort of myocardial bridge patients within our clinical program and look forward to reporting these data soon. We are hopeful that this review serves to inspire further research into the genomic associations behind myocardial bridge development and spur further interest in this important congenital coronary anomaly.

## 6. Limitations

This review does not claim a causal role for any gene found to be associated with myocardial bridge development, as this would require the extensive characterization of how each variant affects protein products and eventual phenotype development. Rather, we hope this review will serve as a comprehensive resource of the current genomic associations that may stimulate further hypothesis-driven research into the genomic pathways responsible for myocardial bridge development. We believe that further detailed mechanistic studies regarding the candidate genes of interest will help to better define the role of each mutation and their subsequent phenotypic effects. Further, though we used significant publicly available publication databases including Pubmed, CINAHL, EMBASE, and Cochrane, we acknowledge that there may be other relevant papers that were not found in these selected databases.

## Figures and Tables

**Table 1:** Four databases were searched using the search terms found in the table. The databases included PubMed, CINAHL, Embase, and Cochrane. Eleven relevant articles were located, including duplicates.

Database	Search Terms	Number of Results	Number of Relevant Articles
PubMed	“Myocardial bridging or bridging” AND “gene or genomics or allele”	14	4
CINAHL	“Myocardial bridging or bridging” AND “gene or genomics or allele”	1	1
Embase	“Myocardial bridging or bridging” AND “gene or genomics or allele”	38	6
Cochrane	“Myocardial bridging or bridging” AND “gene or genomics or allele”	0	0

**Table 2:** Of the eight eligible sources, six were case reports. These reports demonstrated 10 different genetic variations associated with myocardial bridging in patients that ranged from 9 months to 50-years-old.

Author	Subject Age (Years)	Gender	Mutation	Cause of Death
Liu, Y. et al.	50	Female	DES (E434K)	N/A
Sun, Y. et al.	12	Female	FBN1 (I1175M)	N/A
Brancaccio, M. et al.	19	Male	COMMD10; MACROD2	N/A
Grassi, S. et al.	20	Male	SLMAP	Sudden cardiac death
Greenway, S. et al.	9 months	Female	MYH7 (A1157G)	Restrictive cardiomyopathy
Grassi, S. et al.	7	Female	DPP6 (A714T); MYH7 (A862V); SCN2B (E31D); NOTCH1 (R2313Q)	Sudden cardiac death

**Table 3:** Genetic variants (single nucleotide variants) associated with myocardial bridging and candidate gene likelihood.

Genes	Mutations	Candidate Gene
*MYH7*	A1157G	Yes
*DPP6*	A714T	No
*SCN2B*	E31D	Yes
*NOTCH1*	R2313Q	Yes
*SLMAP*	S200L	No
*COMMD10*	Intron 5 CNV	Yes
*MACROD2*	CNVs	Yes
*FBN1/FBN2*	I1175M	Yes
*DES*	E434K	No
*PRKAG2*	R302Q	No

## Data Availability

Data discussed in this report are publicly available as noted in references. No original data is reported.

## Abbreviations

Myocardial bridging (MB), right coronary artery (RCA), left anterior descending (LAD), left circumflex (LCX), congenital coronary vascular anomalies (CCVAs), computed tomography (CT), hypertrophic cardiomyopathy (HCM), restrictive cardiomyopathy (RCM), sudden cardiac death (SCD), variant of unknown significance (VUS), small interfering RNA (siRNA), array-comparative genomic hybridization (array-CGH), copy number variation (CNV), cullin-RING E3 ubiquitin ligase complexes (CRL), nuclear factor kappa-light-chain-enhancer of activated B cells (NFK-ß), congenital heart disorders (CHD), Wolff-Parkinson-White (WPW), microRNA (miRNA).
